# Calcium-loaded acylated segment controls membrane penetration capacity of repeats-in-toxin cytolysins

**DOI:** 10.1016/j.jbc.2026.113079

**Published:** 2026-04-27

**Authors:** Jiri Masin, Adriana Osickova, Zuzana Kalaninova, Petr Man, Ladislav Bumba, Sascha Vatic, Petr Novak, Michaela Buresova, Anna Lesniak, Joana Filipa Tinoco Marçal, David Jurnecka, Humaira Khaliq, Peter Sebo, Radim Osicka

**Affiliations:** 1 Institute of Microbiology of the Czech Academy of Sciences, Prague, Czech Republic; 2 Faculty of Sciences, Charles University, Prague, Czech Republic

**Keywords:** calcium binding sites, folding, RTX toxin, adenylate cyclase toxin, alpha hemolysin, hydrogen/deuterium exchange, membrane interaction, acylated segment, mass spectrometry

## Abstract

Loading of calcium ions into the numerous carboxy-proximal binding sites in the repeats-in-toxin (RTX) domains drives the cooperative and vectorial folding of RTX β-roll structures involved in cell binding and membrane penetration of RTX cytolysins. Two additional binding sites for calcium ions, coordinated by the side chains of residues D880, D918, and N936, were identified in the structure of the acylated cap of the RTX domain of *Bordetella pertussis* adenylate cyclase toxin (CyaA). We show that this calcium-binding structure plays a key role in membrane insertion of the toxin. An N936L residue substitution did not impact toxin acylation or CR3 receptor binding but disrupted the calcium-driven folding of the acylated segment and ablated the membrane penetration capacity of the toxin. Similarly, substitution of the corresponding D639 residue of *Escherichia coli* α-hemolysin abolished its cytolytic capacity. Moreover, disruption of the β-turn structures in the calcium-binding sites of the acylated segment of CyaA (G934L) and α-hemolysin (G637L) strongly impaired the cytotoxic capacities of both toxins. On the contrary, a D880L substitution yielded a CyaA toxin with an enhanced CR3-independent cell penetration and pore-forming capacity. Hydrogen/deuterium exchange probing revealed that the D880L substitution altered the fold of the acylated segment and the interaction of its two acylated β-hairpins. Hence, loading the calcium-binding sites in the acylated segment controls the structure and rules the interaction and the functional cooperation of the two acylated β-hairpins that facilitate penetration of the CyaA polypeptide into the cell membrane.

Repeats-in-toxin (RTX) toxins form a broad family of pore-forming cytolysins secreted by many Gram-negative bacteria. These toxins facilitate infections by subverting and destabilizing host cellular barriers (1, 2). Each RTX toxin consists of four conserved structural and functional portions: (i) a hydrophobic N-terminal pore-forming domain with several predicted transmembrane α-helices, (ii) an acylated segment with two conserved lysine residues that are modified at the ε-amino group by a co-expressed toxin-activating acyltransferase, converting the inactive RTX protoxin into the active RTX toxin, (iii) a C-terminal RTX domain consisting of various numbers of the aspartate- and glycine-rich nonapeptide repeats, forming Ca^2+^-loaded β-roll structures, and (iv) an unprocessed C-terminal secretion signal sequence required for excretion of the toxin from the bacterial cytosol directly into the external milieu through the type I secretion system. Upon interaction with host cells, RTX toxins penetrate the plasma membrane of cells and form cation-selective pores that permeabilize cells for ion fluxes, eventually provoking colloid-osmotic (oncotic) cell lysis (3, 4, 5, 6, 7). On top of this, the bifunctional adenylate cyclase toxin (CyaA, Fig. 1*A*) of *Bordetella pertussis* also possesses a unique N-terminal adenylate cyclase (AC) enzyme fused to the C-terminal RTX cytolysin part (8, 9). The AC domain is translocated into cell cytosol (10, 11), where it is activated by calmodulin and catalyzes an unregulated conversion of intracellular ATP to cAMP (Fig. 1*B*), a key signaling molecule. Supraphysiological levels of cytosolic cAMP then disrupt cellular signaling pathways, ablate bactericidal functions of sentinel phagocytes and eventually provoke their epigenetic reprogramming and apoptotic death (9, 12, 13, 14, 15). CyaA exhibits a paradigm-shifting mechanism of action by its ability to deliver an enzymatic AC domain into target cell cytosol directly across the cellular plasma membrane, without endocytosis or vesicular trafficking. The AC translocation process is rapid and independent of membrane permeabilization by the oligomeric CyaA pore (Fig. 1*B*) (16). This makes CyaA a powerful model system for addressing fundamental questions on Ca^2+^-driven protein folding and conformational switching, or membrane insertion and translocation of large acylated protein domains across membranes.

**Figure 1:**
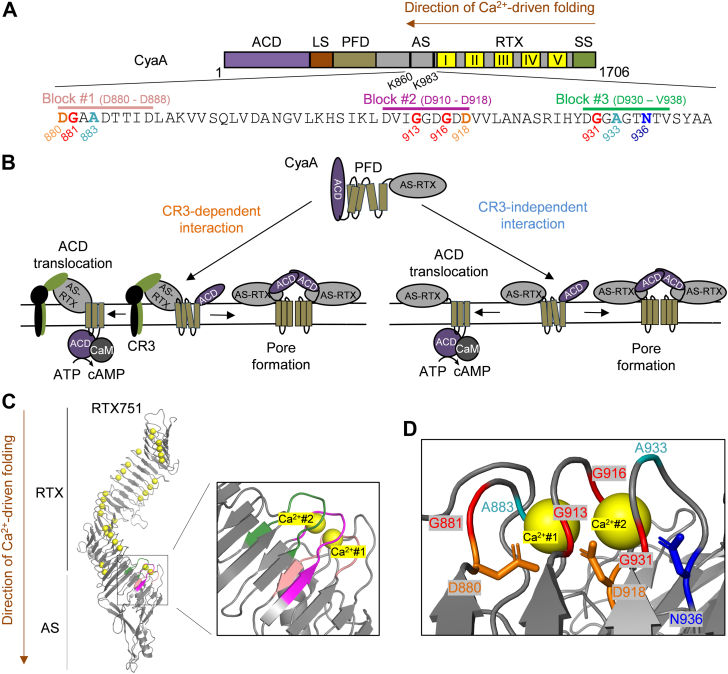
**Depiction of the domain architecture of CyaA and schematic model of CyaA action.***A*, the N-terminal AC enzyme domain, ACD; linker segment, LS; pore-forming domain, PFD; acylated segment, AS; RTX domain, RTX; secretion signal, SS. . The Gly and Asp/Asn-rich nonapeptides within the acylated segment are highlighted in *salmon* (block #1, residues D880 to D888), *violet* (block #2, residues D910 to D918), and *green* (block #3, residues D930 to V938). Ca^2+^ ion (#1) is coordinated by the side chains of residues D880 and D918 (both highlighted by *orange*) and the main chain carbonyls of residues G881 (*red*), A883 (*cyan*), and G913 (*red*). The other Ca^2+^ ion (#2) is coordinated by the side chains of residues D918 (*orange*) and N936 (*blue*), along with the main chain carbonyls of residues G916 (*red*), G931 (*red*) and A933 (*cyan*). Two acylated lysine residues (K860 and K983) are highlighted. The direction of Ca^2+^-driven folding is indicated by a *brown arrow*. *B*, CyaA predominantly targets host myeloid phagocytes that express the β_2_ integrin CR3, but with lower efficacy it also interacts with cells not expressing CR3. The cell-invasive and pore-forming activity of CyaA appears to be independent processes that occur in parallel within the target cell membrane (16, 63, 64). According to the current model, two distinct CyaA conformers penetrate the membrane. One acts as a translocation precursor, delivering the AC domain across the lipid bilayer. The other serves as a pore precursor, oligomerizing into CyaA pores. CaM: calmodulin; CR3: CyaA integrin receptor. *C*, RTX751 fragment displayed using PyMol (PDB 7USL (33)). Nonapeptide sequences involved in binding of Ca^2+^ ions #1 and #2 are highlighted in *salmon* (block #1), *violet* (block #2), and *green* (block #3). Ca^2+^ ions are shown as *yellow spheres*. *D*, residues involved in coordination of Ca^2+^ ions #1 and #2 through their side chains or main chain carbonyls are highlighted. The color coding of the residues coordinating Ca^2+^ ions is the same as in panel 1A. CyaA, adenylate cyclase toxin.

Several RTX toxins have been shown to specifically bind β_2_ integrins of leukocytes (1). CyaA binds highly selectively the α_M_ (CD11b) subunit of the α_M_β_2_ integrin serving as complement receptor 3 (CR3, Mac-1, or CD11b/CD18) of myeloid phagocytic cells (17, 18). Alternatively, the shared β subunit (CD18) of the β_2_ integrin family is less selectively recognized by some other RTX cytolysins (1, 10, 19, 20). Nevertheless, with varying efficacy, the RTX toxins can also promiscuously bind and penetrate a broad spectrum of cell types that do not express β_2_ integrin molecules. Unlike cytolytic RTX toxins, RTX adhesins, a related subgroup within the larger RTX protein family, represent very large cell-surface adhesion proteins of Gram-negative bacteria that facilitate attachment and biofilm formation *via* extensive tandem repeat regions coordinated by Ca^2+^ ions (21, 22). However, Ca^2+^-binding β-rich motifs are not unique to the RTX proteins or the even larger multifunctional autoprocessing RTX toxins (23, 24). Analogous parallel β-roll architectures have been identified in Proline-Glutamate Polymorphic GC-Rich Sequences proteins of *Mycobacterium tuberculosis*. In addition, other protein families, such as βγ-crystallins, employ β-rich folds for Ca^2+^ coordination and structural stabilization (25, 26).

The RTX domain of CyaA has been shown to be intrinsically disordered in the absence of Ca^2+^ ions, such as inside bacterial cytoplasm, and to fold cooperatively upon Ca^2+^ binding in a process that is essential for RTX toxin secretion, stability and function (27, 28, 29). The Ca^2+^-binding RTX nonapeptide repeats of all RTX toxins share the consensus motif G-G-X-G-X-D/N-X-U-X, where U represents the hydrophobic residue valine, leucine, or isoleucine and X represents any residue (30). The number of tandemly arranged repeats varies from 10 to over 40 in different RTX toxins and their exact number depends on how strictly the consensus motif is followed (2, 31). Early on, the crystal structures of the smaller RTX domains were solved for the alkaline RTX protease of *Pseudomonas aeruginosa* and the RTX lipase from *Serratia marcescens*. More recently, the structure of the much larger RTX domain of CyaA could be resolved using the Cryo-EM technology (30, 32, 33, 34, 35). This revealed that the first six residues of the nonapeptide repeat (G-G-X-G-X-D/N) form a β-turn involved in Ca^2+^ ion binding and the last three residues (X-U-X) form a short β-strand. The Ca^2+^ ions bind between two adjacent β-turns, primarily through the main chain carbonyl groups of the glycine residues and the negatively charged carboxyl groups of the aspartate residues, which form a hexa-coordinated Ca^2+^ binding site. Binding of Ca^2+^ ions triggers folding of the five RTX blocks into parallel Ca^2+^-loaded β-roll structures, with the folding signal propagating vectorially from the C-terminus towards the N-terminus of the RTX domain (27, 32, 36, 37, 38). The resulting biologically active toxin structure also comprises an acylated segment that forms a β-roll extension of the RTX domain and harbors two β-hairpins with two conserved lysine residues (K860 and K983 in CyaA, Fig. 1*A*) at their tips. Fatty-acylation of the ε-amino groups of these lysine residues by a co-expressed toxin-activating acyltransferase then activates the RTX protoxins by conferring on them the membrane-penetrating capacity (7, 39, 40, 41). Despite the functional importance of the acylated segment, its Ca^2+^-dependent structural organization and the mechanism by which it initiates membrane insertion remain poorly understood.

Here, we identified a new conserved structure in the acylated segments of pore-forming RTX toxins that plays a key role in Ca^2+^-dependent folding and membrane penetration capacity of *B*. *pertussis* CyaA and *Escherichia coli* HlyA.

## Results

### Ca^2+^-binding structures in the acylated segment control membrane penetration of CyaA

**Figure 2:**
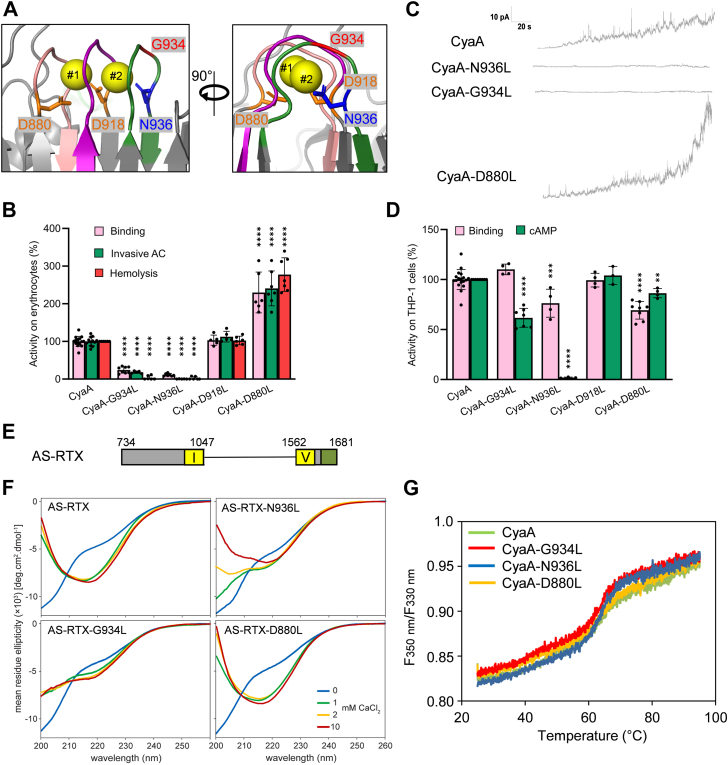
**Substitutions of N936 and G934 affect folding of the acylated segment and cell-penetrating activity of CyaA.***A*, PyMol visualization of a part of the acylated region of RTX751. D880, D918, and N936, which coordinate Ca^2+^ ions #1 and #2 (*yellow spheres*) *via* their side chains are marked in *orange* and blue. G934 involved in formation of β-turn is shown in red. Nonapeptides involved in binding of Ca^2+^ ions #1 and #2 are highlighted in *salmon* (block #1), *violet* (block #2), and *green* (block #3). *B*, cell binding and cell invasive AC activities of CyaA variants were assessed using erythrocytes (5 × 10^8^/ml) incubated with 1 μg/ml of protein in TCN buffer (pH 7.4) for 30 min at 37 °C. Hemolytic activity was assessed on sheep erythrocytes (5 × 10^8^/ml) exposed to 5 μg/ml of the protein for 4 h at 37 °C as A_541_. The activity of WT CyaA was set to 100%. Data represent mean values from at least five independent experiments, each performed in duplicate, using two independent toxin batches. *C*, overall membrane activities of 250 pM CyaA variants on planar lipid membranes. The aqueous phase contained 10 mM Tris-HCl (pH 7.4), 2 mM CaCl_2_, and 150 mM KCl. Experiments were performed at 25 °C under a voltage of -50 mV, and signals were filtered at 10 Hz. *D*, binding of CyaA variants to human THP-1 cells (1 × 10^6^ cells in DMEM) was assessed at 4 °C by measuring cell-associated AC after incubation with 1 μg/ml protein for 30 min. Intracellular cAMP levels were determined in THP-1 cells (1.5 × 10^5^ in DMEM) incubated at 37 °C for 30 min with various CyaA concentrations (250–15.5 ng/ml). Activities are expressed as percentages of WT CyaA activity and represent means ± SD from at least three independent experiments performed in duplicate, using two different toxin preparations. Statistical significance was determined by the one-way ANOVA. ∗∗∗∗, *p* < 0.0001, ∗∗∗, *p* < 0.001, ∗∗, *p* < 0.01. *E*, AS-RTX construct consists of the part of the acylated segment and block I of RTX repeats (residues 735–1047) at the N-terminus and block V of the RTX repeats and part of the secretion signal (residues 1562–1681) at the C-terminus. *F*, far-UV CD spectra of AS-RTX variants (100 μg/ml) refolded from 8 M urea in buffer containing 5 mM Tris-HCl (pH 8.0), 20 mM NaCl, and 12.5 mM imidazole, with CaCl_2_ concentrations ranging from 0 to 10 mM. *G*, typical thermal unfolding curves of Ca^2+^-loaded CyaA and its CyaA-G934L, CyaA-N936L, and CyaA-D880L variants (100 μg/ml in TCN buffer). CyaA, adenylate cyclase toxin.

The sequence of the acylated segment of CyaA harbors three Gly and Asp/Asn rich motifs (blocks #1 to #3; Fig. 1, *A* and *C*) that resemble the Ca^2+^-binding RTX repeats located in the C-terminal region of the toxin. Indeed, the recently solved structure of the RTX751 protein (33) contains two Ca^2+^ ions bound at these sites (Fig. 1*C*). One Ca^2+^ ion (#1) is coordinated by the side chains of residues D880 and D918 and by the main chain carbonyls of residues G881, A883, and G913. The second Ca^2+^ ion (#2) is coordinated by the side chains of residues D918 and N936 together with the main chain carbonyls of residues G916, G931, and A933 (Fig. 1, *A* and *D*). We hypothesized that Ca^2+^ loading into these sites determines the functional fold of the acylated segment, which plays a key role in the initiation of membrane insertion of the toxin. As the Ca^2+^-driven folding of the RTX domain propagates vectorially from the C-terminal scaffold towards the N-terminal acylated cap (33, 37, 42), we first examined the functional role of the first-folding block #3. This contains the first six residues of the classical nonapeptide RTX repeat unit (G-G-X-G-X-D/N) that form a β-turn involved in Ca^2+^ binding (30, 32). Therefore, the conserved residues G931, G932, G934, and N936 within this β-turn were individually replaced by a bulky hydrophobic leucine side chain, expected to perturb the structure of the Ca^2+^-binding site #2 (Fig. 1, *A* and *D*, and 2*A*) and affect the function of the entire acylated segment. To assess the capacity of CyaA variants to penetrate target membranes, we used human THP-1 cells as a model of myeloid phagocytes that express the CyaA receptor CR3. In parallel, sheep erythrocytes lacking CR3 were used as a model of non-myeloid target cells. As shown in Figure 2*B*, the purified CyaA-N936L variant exhibited a significantly reduced capacity to bind and penetrate the CR3-deficient erythrocytes. Consequently, the CyaA-N936L toxin failed to deliver its AC domain into erythrocyte cytosol and failed to penetrate and permeabilize erythrocyte membrane through formation of hemolytic pores (Fig. 2*B*). Consistently, the N936L substitution almost completely abolished the capacity of the toxin to penetrate and form pores in naked artificial lipid bilayers with an imposed membrane potential (Fig. 2*C*). However, once rare individual pores formed, they exhibited single-pore conductance and lifetime values similar to those of the pores formed by intact CyaA (Fig. S1). This loss of function was not due to impaired acylation, as the N936L substitution did not affect the recognition and acylation of the K860 and K983 residues by the acyltransferase CyaC during toxin production in *E. coli* cytosol (Table 1). In addition, the observed functional defect was most likely not due to misfolding of the RTX domain, as the CyaA-N936L protein bound the CR3 receptor of THP-1 monocyte cells nearly as efficiently as the intact CyaA (Figs. 2*D* and S2). Hence, upon CR3-mediated positioning toward the cell membrane, the CyaA-N936L molecule could still insert its acyl chains into the outer leaflet of THP-1 monocyte cell membrane to form a stable complex with CR3 (43, 44). Nevertheless, the CyaA-N936L protein was unable to translocate its AC domain across the membrane into the CR3-expressing THP-1 cells to increase cytosolic cAMP levels (Fig. 2*D*). Hence, the N936L substitution selectively impaired the subsequent step of CyaA action that follows receptor binding and yields insertion of the hydrophobic pore-forming domain of the toxin molecule into cell membrane and translocation of the N-terminal AC enzyme polypeptide into cell cytosol (Fig. 2*D*). Proper coordination of the Ca^2+^ ion #2 by the asparagine side chain of the N936 residue thus appears to play a crucial role in the membrane insertion-promoting function of the acylated segment of CyaA.

**Table 1:** Acylation status of adenylate cyclase toxin and α-hemolysin proteins

Protein^a^	Modification	K860^b^	K983^b^
CyaA	non-modified	16.1 ± 2.4	6.0 ± 0.5
	C16:0	40.6 ± 4.7	25.8 ± 1.5
	C16:1	34.6 ± 2.4	61.8 ± 1.1
	C18:1	8.5 ± 1.4	5.3 ± 0.7
CyaA-D880L	non-modified	83.0 ± 3.3	4.9 ± 0.9
	C16:0	6.2 ± 1.1	26.2 ± 1.7
	C16:1	10.7 ± 2.3	61.6 ± 2.9
	C18:1	-	5.7 ± 1.1
CyaA-G934L	non-modified	24.8 ± 5.6	8.3 ± 1.3
	C16:0	32.0 ± 5.6	23.3 ± 2.1
	C16:1	35.1 ± 7.5	63.5 ± 2.9
	C18:1	7.9 ± 1.5	3.5 ± 0.9
CyaA-N936L	non-modified	17.4 ± 2.6	6.4 ± 0.5
	C16:0	32.0 ± 6.4	26.9 ± 0.5
	C16:1	42.4 ± 4.5	60.2 ± 0.8
	C18:1	8.0 ± 1.8	5.1 ± 0.9


Protein^a^	Modification	K564^b^	K690^b^
HlyA	non-modified	6.5 ± 1.5	-
	C14:0	38.7 ± 5.5	69.5 ± 0.6
	C14:0-OH	40.5 ± 8.3	24.9 ± 0.5
	C14:1	14.1 ± 11.7	2.4 ± 0.3
HlyA-G637L	non-modified	6.1 ± 0.6	-
	C14:0	39.5 ± 2.1	65.7 ± 3.3
	C14:0-OH	41.9 ± 3.8	27.7 ± 2.7
	C14:1	9.7 ± 2.8	2.1 ± 0.3
HlyA-D639L	non-modified	7.7 ± 2.1	-
	C14:0	48.7 ± 2.5	70.3 ± 4.1
	C14:0-OH	39.0 ± 3.4	23.8 ± 3.6
	C14:1	4.5 ± 0.9	2.6 ± 0.2

a Proteins were expressed in the *E. coli* strain XL-1, purified and analyzed as described in Experimental procedures.

b The percentage distribution of fatty acylation at K860 and K983 (for CyaA) or at K564 and K690 (for HlyA) ± SD is shown. Two independent toxin preparations were analyzed.

In contrast, the CyaA-G934L variant retained an intact CR3-dependent cell binding capacity and its specific ability to translocate the AC domain into THP-1 cells was reduced by approximately 40% (Figs. 2*D* and S2). However, the toxin bound only poorly to erythrocytes lacking the CR3 receptor, and its AC translocation and pore-forming activities on erythrocytes and in artificial lipid bilayers were almost completely abolished (Fig. 2*B* and 2*C*). Similar to the CyaA-N936L toxin, the rare individual pores formed by CyaA-G934L exhibited single-pore conductance and lifetime values similar to those of the pores formed by intact CyaA (Fig. S1). This indicates that the introduced substitutions (N936L and G934L) strongly reduced the propensity of the mutants to form functional pores in the lipid bilayer while having no impact on the individual pore characteristics. Thus, in the absence of positioning of the toxin molecule on the membrane through interaction with the CR3 receptor, the specific membrane penetration capacity of the CyaA-G934L variant was significantly reduced.

Similar functional defects were observed when the residues G934 and N936 were replaced by a proline residue to disrupt the β-turn structure. As shown in Fig. S3, the G934P and N936P substitutions reduced toxin activities to a similar extent as the corresponding leucine substitutions (G934L and N936L), confirming a key functional role of both residues.

In contrast, leucine substitutions of the G931 and G932 residues in the Ca^2+^-binding block #3 (Fig. 1*A*) had negligible, if any, impact on the binding and cell penetration capacities of CyaA on both erythrocytes and THP-1 cells (Fig. S4). The very C-proximal G934 residue of block #3 thus appears to play an important role in formation of the β-turn structure, whereas the subsequently folding G931 and G932 residues are of lower importance for the vectorial Ca^2+^-driven folding that proceeds from the C-terminal end towards the N-terminus (37). This outcome demonstrates that not all glycine residues within the same Ca^2+^-binding RTX loop are functionally equivalent and the phenotype of their substitutions is position-specific and not a simple consequence of residue replacement.

Intriguingly, a leucine substitution of D918 of Ca^2+^-binding block #2, engaged in coordination of Ca^2+^ ions #1 and #2 *via* its negatively charged side chain carboxyl group (Figs. 1*D* and 2*A*), had no impact on the cell binding and penetrating capacity of the CyaA-D918L toxin (Fig. 2, *B* and *D*). Hence, the D918 carboxyl does not appear to play an essential role in the Ca^2+^-coordinating structure. Indeed, as shown in Fig. S5, simultaneous leucin substitutions of up to three charged Asp residues at the positions 915, 917, and 918, likely disrupting the integrity of the entire Ca^2+^-binding block #2, was required to ablate the functional structure as well as the cell penetrating and cell permeabilizing activity of CyaA. Unexpectedly, the substitution of the D880 residue, coordinating *via* its side chain carboxyl the Ca^2+^ ion #1, significantly increased the capacity of the CyaA-D880L toxin to penetrate erythrocyte or artificial planar lipid bilayer membranes devoid of the CR3 receptor (Fig. 2, *B* and *C*). At the same time, the specific CR3-dependent membrane penetration capacity of the CyaA-D880L toxin on THP-1 cells was only slightly decreased (Figs. 2*D* and S2). Moreover, the properties of individual pores formed by the membrane-inserted CyaA-D880L toxin in bilayer membranes, such as the most frequent conductance and lifetime, remained the same as that of intact CyaA (Fig. S1). The D880L substitution thus highly selectively enhanced the CR3-independent membrane penetration and AC translocation and pore forming propensity of CyaA.

Using CD spectroscopy, we next assessed the structural impact of the N936L, G934L, and D880L substitutions on the fold of the acylated domain. For this purpose, we designed an AS-RTX construct, in which the acylated segment (AS, residues 734–1047) was linked with a hybrid RTX block I-V C-terminal folding scaffold (residues 1562–1681) to form a Ca^2+^-loaded β-roll structure propagating the folding signal into attached the N-terminal acylated segment (Fig. 2*E*). Indeed, in the absence of Ca^2+^ ions, the AS-RTX, AS-RTX-N936L, AS-RTX-G934L, and AS-RTX-D880L proteins displayed typical CD spectra of unfolded polypeptides (Fig. 2*F*). In contrast, upon titration with Ca^2+^ ions the spectra of all four proteins contained the characteristic negative peak at 218 nm corresponding to formation of Ca^2+^-loaded RTX β-rolls (Fig. 2*F*). The D880L substitution did not affect the Ca^2+^-dependent folding of the AS-RTX construct (Fig. 2*F*, lower right panel), which at increasing Ca^2+^ concentration yielded the same spectra as the intact AS-RTX. In contrast, the CD spectra of AS-RTX-G934L and AS-RTX-N936L exhibited significantly lower intensities of the band at 218 nm, even at a 10 mM Ca^2+^ concentration, indicating that the N936 and G934 residues are critical for Ca^2+^-driven folding of the β structures of the acylated segment. While the N936 residue directly coordinates the Ca^2+^ ion #2, G934 does not participate in Ca^2+^ coordination (Fig. 2*A*) and its effect therefore most likely arises from perturbation of the Ca^2+^-binding β-turn structure. Moreover, unlike AS-RTX, the AS-RTX-N936L construct misfolded upon exposure to 1 mM, 2 mM, or 10 mM Ca^2+^. Its CD spectrum exhibited an increase of mean residue ellipticity between 200 and 210 nm, with a negative peak around 208 nm, indicating the formation of α-helical structures (Fig. 2*F*). In line with the disruption of a functional structure of the acylated segment upon introduction of the N936L substitution, the full-length CyaA-N936L variant then failed to penetrate erythrocyte and THP-1 monocyte membranes even at a saturating 10 mM Ca^2+^ concentration (Fig. S6). However, nano differential scanning fluorimetry (nanoDSF) examination did not reveal altered thermal melting behaviors of the Ca^2+^-loaded full-length CyaA-N936L and CyaA-G934L compared to intact CyaA (Fig. 2*G* and Table S1). This suggests that the N936L and G934L substitutions did not alter the overall stability of full-length CyaA.

### Disruption of a corresponding Ca^2+^-binding structure in the acylated segment of α-hemolysin (HlyA) ablates its cytolytic activity

**Figure 3:**
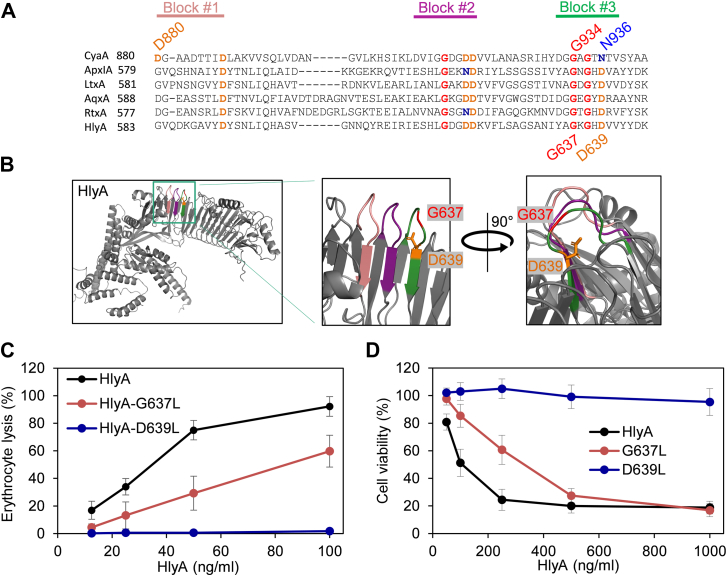
**Disruption of a corresponding Ca^2+^-binding structure ablates the cytolytic activity of HlyA.***A*, clustalW alignment of partial sequences from the acylated regions of RTX toxins. CyaA, *Bordetella pertussis* (UniProtKB P0DKX7); ApxIA, *Actinobacillus pleuropneumoniae* (UniProtKB P55128); LtxA, *Aggregatibacter actinomycetemcomitans* (UniProtKB P16462); AqxA, *Actinobacillus equuli* (UniProtKB Q8KWZ9); RtxA, *Kingella kingae* (UniProtKB A0A1X7QMH9); HlyA, *Escherichia coli* (UniProtKB A0A4Z0T8K2). The sequences of Gly and Asp/Asn-rich nonapeptides within the acylated domain are highlighted in *salmon* (block #1), *violet* (block #2), and *green* (block #3). *B*, AlphaFold model of the part of the acylated segment of HlyA, highlighting aspartate residue 639 (*in orange*) potentially involved in Ca^2+^ ion coordination, and glycine 637 (*in red*) forming a β-turn structure. The sequences of Gly and Asp/Asn-rich nonapeptides within the acylated domain are highlighted in *salmon* (block #1), *violet* (block #2), and *green* (block #3). *C*, sheep erythrocytes (5 × 10^8^/ml) were exposed to HlyA variants at 37 °C, and hemolytic activity was quantified at A_541_ after 15 min. The results represent average values from four independent experiments performed in duplicate, using two independent toxin preparations. *D*, the viability of THP-1 cells (1.5 × 10^5^/well in DMEM) was assessed after 2 h of exposure to the HlyA variants at 37 °C. Data represent mean values from three independent experiments, each performed in duplicate using two different HlyA batches. CyaA, adenylate cyclase toxin; HlyA, α-hemolysin.

Formation of similar Gly- and Asp/Asn-rich Ca^2+^-binding structures can be also predicted in several other RTX cytolysin molecules, such as the *E. coli* α-hemolysin HlyA (Figs. 3, *A* and *B*, and S7*A*). Therefore, we examined the cytolytic activity of a HlyA-D639L variant in which the D639 residue, corresponding to N936 of CyaA and predicted to coordinate a Ca^2+^ ion, was replaced by a leucine residue. As shown in Figure 3*C*, the D639L substitution ablated the hemolytic activity of the fully acylated HlyA-D639L protein (Table 1) and strongly impacted its cytotoxic activity towards THP-1 cells that express the CD18 subunit of β_2_ integrins bound by HlyA (Fig. 3*D*). Moreover, the G637L substitution in HlyA, corresponding to the G934L substitution in CyaA, strongly affected the specific cytolytic and cytotoxic activity of HlyA (Fig. 3, *C* and *D*). Hence, the G637 and D639 residues appear to play the same structural role in HlyA as the G934 and N936 residues of CyaA. As in CyaA, the loading of the predicted Ca^2+^-binding site(s) in the corresponding acylated segment would then play a role also in initiation of membrane penetration of HlyA.

### Alteration of the Ca^2+^-binding structure alters the interaction of the acylated β-hairpins and enhances CR3-independent membrane penetration of CyaA

**Figure 4:**
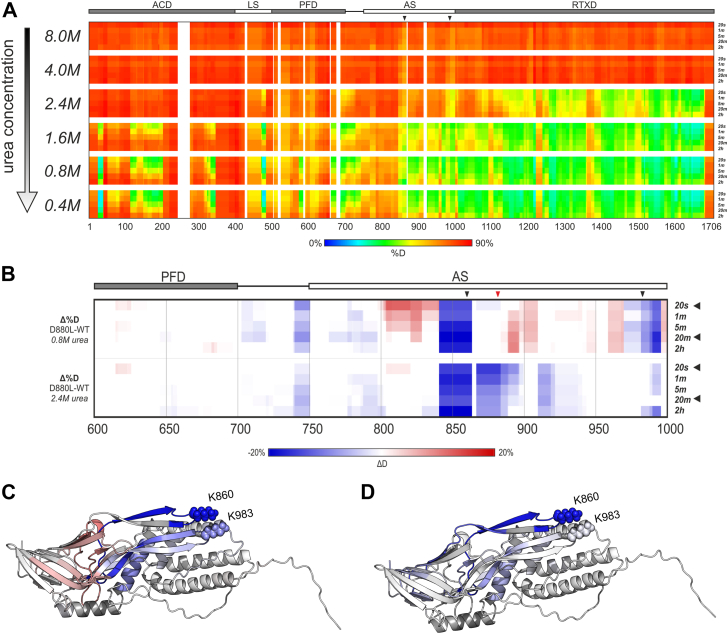
**D880L substitution alters the accessibility of the acylated segment of CyaA to deuteration.***A*, folding of WT CyaA, initiated from the C-terminus, induced by lowering the urea concentration and addition of Ca^2+^ ions monitored by hydrogen/deuterium exchange mass spectrometry. Obtained deuteration profiles were visualized using rainbow heat maps. Toxin domains and positions of acylation sites (*black arrowheads*) are annotated above the heat map. Regions not covered by hydrogen/deuterium exchange mass spectrometry data are represented by white color. *B*, heat maps illustrate differences in deuterium incorporation between WT CyaA and the D880L mutant at final urea concentration of 0.8 M and 2.4 M. The deuteration level of intact CyaA was subtracted from that of the CyaA-D880L variant. Color coding reflects changes in deuterium uptake: *blue* indicates protection (lower deuteration), *white* indicates no change, and *red* indicates deprotection (*higher deuteration*). Domains, acylation sites (black arrowheads), and D880 position (red arrowhead) are annotated above the heat maps. CyaA residues 600 to 1000 are colored according to Δ%Dav at 0.8 M urea (*C*) and 2.4 M urea (*D*). Δ%Dav is defined as the average of the three time points showing the largest differences between the two protein states (WT and D880L). This representation condenses the full hydrogen/deuterium exchange kinetic data into a single value, facilitating visualization in a structural context. The same color scale is used as for panel B. Heat maps for full-length CyaA are provided in the Supporting Information (Fig. S8*B*). CyaA, adenylate cyclase toxin.

The CyaA-D880L toxin variant exhibited an intriguingly increased CR3-independent capacity to penetrate cellular and artificial membranes (Fig. 2, *B* and *C*). Moreover, the thermal stability of the Ca^2+^-loaded full-length CyaA-D880L was comparable to that of intact CyaA (Fig. 2*G* and Table S1), suggesting that the D880L substitution did not alter the overall stability and structure of the protein. Therefore, we examined the Ca^2+^-driven formation of the acylated segment structure of CyaA-D880L by hydrogen/deuterium exchange (HDX). We first monitored the dynamics of Ca^2+^-mediated folding to assess the stability of CyaA in its folded state. Fully denatured CyaA in high urea was subjected to Ca^2+^-induced folding while the urea concentration was gradually reduced (Fig. 4*A*). These data corroborate previous findings (42) demonstrating that CyaA folding initiates at the C-terminus and then sequentially propagates toward the N-terminal region as the urea concentration decreases (Fig. 4*A*). The major folding transition occurred between 4.0 M and 2.4 M urea, followed by compaction of the central and N-terminal regions between 2.4 M and 1.6 M urea. Folding of the N-terminal region was completed between 1.6 M and 0.8 M urea. Further reduction of urea to 0.4 M did not induce additional structuring of the RTX domain and the acylated segment, and only minor stabilization was observed in the N-terminal regions (Fig. S8*A*). Based on these observations, we next examined differences at 0.8 M urea and additionally at 2.4 M urea to probe the potential effects of the D880L substitution on CyaA stability. As illustrated in Fig. S8*B*, in the presence of 0.8 M urea, the structure and fold of the AC domain, of the AC-Hly linker segment, and of the RTX domain remained unaffected by the D880L substitution. However, an increased deuteration of the backbone amide hydrogens of the CyaA-D880L protein (Δ%D D880L-WT) was observed in the regions spanning the residues ∼570 to 580, ∼800 to 840, ∼880 to 970, and ∼995 to 1000. In contrast, the segments ∼700 to 800, ∼840 to 860, and ∼970 to 995, including the β-hairpins bearing the acylated K860 and K983 residues, were less accessible to deuteration in the CyaA-D880L protein than in WT CyaA (Fig. 4, *B* and *C*). These findings suggested that the D880L substitution altered the folding of the structures throughout nearly the entire acylated segment.

To corroborate this, the HDX analysis was performed at an increased concentration of 2.4 M urea to probe the folding of the acylated segment of the CyaA-D880L protein under mildly denaturing conditions. As shown in Fig. S8*B*, the D880L substitution did not impact on the folding of the structures of the AC domain, linker segment and hydrophobic domain (white color). Moreover, the extent of deuteration of the CyaA regions that exhibited increased accessibility already in 0.8 M urea did not differ between the WT and D880L CyaA proteins treated in 2.4 M urea. However, the presence of 2.4 M urea revealed a difference between the WT and D880L CyaA proteins in the accessibility to deuteration of the structures around the acylated sites of the acylated segment and the RTX domain (residues ∼1000–1060). These structures remained relatively less accessible to deuteration in the CyaA-D880L protein, compared to WT CyaA, indicating an increased resistance of those structures of the CyaA-D880L protein to relaxation under mildly denaturing conditions (Figs. 4, *B* and *D*, and S8).

## Discussion

**Figure 5:**
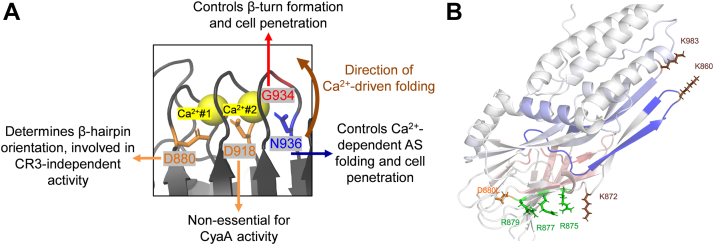
*A*, **the role of residues coordinating two Ca^2+^ ions in folding and cytotoxic activity of CyaA.** Residues D880, D918, and N936 coordinating Ca^2+^ ions #1 and #2 (*yellow spheres*) are marked in *orange* and *blue*, respectively, the G934 is marked in *red*. The direction of Ca^2+^-driven folding is indicated by a *brown arrow*. *B*, visualization of RTX751 in PyMol (PDB 7USL). Aspartate D880 is shown in *orange*, lysine residues K872, K983, and K860 are in *brown*, and arginine residues R875, R877, and R879 are in *green*. Changes in deuteration are represented by a color gradient: *blue* indicates protection (*lower deuteration*), *white* indicates no change, and *red* indicates deprotection (*higher deuteration*). CyaA, adenylate cyclase toxin.

This work identifies a previously unrecognized structural motif in the acylated segments of pore-forming RTX toxins that plays an essential role in their Ca^2+^-dependent folding and determines the membrane penetration capacity of the CyaA toxin (Fig. 5*A*). Indeed, the recently solved structure of the CyaA RTX751 fragment revealed two adjacent binding sites for two Ca^2+^ ions #1 and #2 (Fig. 1) within the acylated extension that caps the RTX domain (33). We show that substitution of its N936 residue involved in coordination of the Ca^2+^ ion #2, results in misfolding of the acylated segment and provokes a loss of the cell binding and membrane penetration capacity of CyaA. In striking contrast, replacement of the D880 residue, involved in coordination of the Ca^2+^ ion #1, yielded a highly selective twofold enhancement of the CR3-independent cell binding and membrane penetration capacity of CyaA (Fig. 5*A*). Such strikingly opposing impacts of perturbations at the two Ca^2+^-binding sites indicate that these structures act as a regulatory 'calcium lock' that dictates the Ca^2+^-dependent folding and orientation of the acylated segment, thereby directly controlling the membrane insertion step that precedes pore formation and AC translocation.

The acylated segment that N-terminally caps the RTX domain is a highly conserved and functionally important domain of the pore-forming RTX toxins. It harbors the two conserved lysine residues modified by fatty acyl chains involved in membrane penetration of the RTX toxins (7, 39, 45, 46). The portion between the two acylation sites includes three Gly- and Asp/Asn-rich nonapeptide motifs (Fig. 1*A*) that resemble the C-terminal RTX repeats. Importantly, folding of the RTX domain with its N-terminal acylated cap is initiated by binding of Ca^2+^ ions within the C-terminal scaffold structure of the RTX domain. The folding signal then proceeds vectorially, towards the N-terminus, following a sequential and highly cooperative templating-like mechanism that yields formation of five consecutive β-roll structures loaded by ∼40 Ca^2+^ ions (27, 32, 33, 35, 37, 43, 47, 48). It is thus plausible to assume that folding of the rest of the RTX domain, preceding the folding of the acylated segment, was not perturbed by the N936L substitution located far downstream from the folding initiation site. Indeed, the double acylated CyaA-N936L toxin variant retained a high capacity to engage CR3 through its properly folded CR3-binding structure formed by the RTX blocks I to III and their L1 and L2 linkers (17, 18, 33, 49). However, the CR3-mediated association of the CyaA-N936L protein with the cell membrane was unproductive, as the specific capacity of the CyaA-N936L protein to translocate its N-terminal AC enzyme domain into the cytosol of THP-1 monocytes was almost nil (Fig. 2*D*). It appears, hence, that replacement of the Ca^2+^ ion #2-coordinating asparagine side chain at position 936 (Fig. 2*A*) by the bulky aliphatic side chain (N936L) disrupted propagation of the Ca^2+^-driven C- to N- vectorial folding signal beyond the residue 936. This would prevent proper formation of the two binding sites for Ca^2+^ ions #2 and #1 and consequently yield misfolding of the N-terminal portion of the acylated segment (Fig. 2*F*). Consequently, the acylated segment would fail to adopt the functional conformation required for insertion of the hydrophobic translocon domain of the toxin into cell membrane (10, 50). The misfolding of the N-terminal portion of the acylated segment beyond residue 936 would then affect the formation and positioning of the β-hairpin that bears the acylated K860 lysine residue at its tip. Indeed, we have previously shown that juxtaposition to the two acylated β-hairpins controls the CR3-independent insertion of CyaA into the membrane of erythrocytes (19, 50). In line with this prediction, the capacity of the CyaA-N936L protein to bind and penetrate erythrocytes was nil (Fig. 2*B*). Moreover, a similar phenotype was observed upon replacement of the hydrophobic core-facing aromatic ring of the tyrosine 940 residue, which stabilizes the structure of the L0 linker connecting the acylated segment to the RTX domain and positions the β-hairpin with the acylated K983 residue (33, 51, 52).

Intriguingly, the G934L substitution in the β-turn forming the Ca^2+^-binding structure selectively impaired the CR3-independent cell penetration capacity of the CyaA-G934L toxin. Upon interaction with the α-subunit of the CR3 receptor, which positions the toxin molecule towards cell membrane, the cell penetration capacity of the CyaA-934L toxin was only mildly affected (*c*.*f*. Figs. 2, *B* and *D*). It appears, hence, that the G934L substitution still allowed some binding of the Ca^2+^ ion into the β-turn of the Ca^2+^-binding site #2 and possibly did not perturb the binding of the Ca^2+^ ion #1. This would allow a functional structuring of the acylated segment and penetration of the toxin into cell membrane.

It is worth noting that leucine substitutions were chosen because of the bulky and branched leucine side chain that strongly restricts the conformational freedom of the peptide backbone and is particularly effective in disrupting the formation of β-turns. Hence, while the G934 residue itself does not appear to coordinate the Ca^2+^ ion #2, a large hydrophobic side chain at the position 934 is likely to sterically interfere with the local geometry of the Ca^2+^-binding pocket formed by the neighboring residues. In line with this interpretation, substitution of G934 with a proline residue, also known to disrupt β-turn geometry, yielded a similar phenotype (Fig. S3). This highlights the functional importance of the G934-comprising β-turn structure.

Unexpectedly, the negatively charged side-chain carboxyl of the D918 residue turned out to be only marginally involved in binding of Ca^2+^ ions #1 and #2. The D918L substitution had no impact on cell binding and penetration capacity of the CyaA-D918L toxin. This suggests that stabilization of the Ca^2+^ ions #1 and #2 in the structure of the acylated segment is accomplished primarily by the side chains of the N936 and D880 residues and by the main chain carbonyls of the G881, A883, G913, G916, G931, and A933 residues (33).

More surprisingly, the D880L substitution increased by about two-fold the CR3-unassisted membrane binding, AC domain translocation, and hemolytic activity of CyaA on erythrocytes (Fig. 2*B*). The same was true for the increased overall membrane activity of the CyaA-D880L toxin on planar lipid membranes (Fig. 2*C*). This appeared to be due to increased membrane penetration propensity of the CyaA-D880L toxin, which increased the specific pore-forming activity of the toxin molecule on planar lipid membranes, but did not alter the properties of the formed CyaA pores (Fig. S1).

Intriguingly, the highly active CyaA-D880L toxin was fully acylated at the K983 residue, but about 80% of the toxin molecules remained non-acylated at the K860 residue. Hence, the D880L substitution yielded a mixture of singly and doubly-acylated CyaA-D880L molecules and it is difficult to predict how their coexistence impacted the overall toxin activity in the used assays. Indeed, fatty acyl modification of the K860 and K983 residues was found to modulate folding and stability of CyaA (42) and a single palmitoylation of the K983 residue appears to be necessary and sufficient for productive membrane interaction of the CyaA toxin (44, 50, 53, 54). On the other hand, both the D880L substitution as well as the absence of an acyl group at the K860 residue may individually or jointly affect the fold of the acylated segment structure (42) and may enhance the CR3-unassisted capacity of the CyaA-D880L toxin to penetrate the erythrocyte membrane. However, our earlier work indicated that mono-and bi-acylated CyaA molecules, having the K983 residue acylated, exhibited the same capacity to penetrate erythrocyte membrane and the presence of acyl modification at the K860 residue only down-modulated the pore-forming propensity of the membrane-inserted CyaA molecules (55). Hence, an enhancing impact of the D880L substitution on the functional structure of the acylated segment, rather than the lack of K860 acylation in about half of the produced CyaA-D880L molecules, would represent a more plausible explanation of the enhanced CR3-unassisted membrane binding and penetration propensity of the D880L toxin. Indeed, the residue corresponding to D880 of CyaA is absent in the acylated segments of some pore-forming RTX cytolysins (Fig. 3*A*). For example, in HlyA the Ca^2+^ ion #1 could either be coordinated by other residue(s) than in CyaA, or only a single Ca^2+^ ion may be bound within the structure of the acylated segment of HlyA. While CD spectroscopy and nanoDSF data indicated that the global folding and stability of the CyaA-D880L variant were comparable to that of intact CyaA (Fig. 2, *F* and *G*), the high resolution HDX analysis revealed some more relaxed regions near the introduced D880L substitution in CyaA. At the same time, the D880L substitution yielded a lower deuteration of the N-terminal region of the acylated segment, or of portions of the β-hairpins bearing the acylated K860 and K983 residues at their tips (Fig. 4). Compared to intact CyaA, lower deuteration of specific regions of the acylated segment was apparent irrespectively of the denaturing urea concentration, indicating a possibly altered positioning of the acylated β-hairpins in the CyaA-D880L protein. These structural changes may make some hydrophobic patches of CyaA structure more prone to exposure and may thus enhance the propensity of the toxin to penetrate the cytoplasmic membrane in the absence of CR3-mediated positioning in respect to cell membrane. Moreover, we have recently observed that substitution of any of the outwards-facing positively charged residues in the _872_KQDRWRIRD_880_ cluster flanked by the D880 residue (Fig. 5*B*), yields a similar enhancement of CR3-unassisted membrane penetration capacity of CyaA (Lesniak *et al*. unpublished). This suggests that the structure comprising the D880 residue is somehow involved in fine-tuning of the CR3-unassisted membrane penetration of the CyaA toxin. Conserved blocks of residues forming potential Ca^2+^-binding sites similar to those of CyaA are, indeed, predicted by AlphaFold in the acylated segment structures of other pore-forming RTX cytolysins, such the *E*. *coli* HlyA, *Actinobacillus pleuropneumoniae* ApxIA, or *Kingella kingae* RtxA (Fig. S7). It is thus plausible to assume that these Ca^2+^-binding structures play a similar functional role in Ca^2+^-driven folding of the acylated segments and in membrane penetration of the RTX family cytolysins in general.

## Experimental procedures

### Bacterial strains

*E. coli* XL1-Blue cells (Stratagene) were cultured in Luria-Bertani medium at 37 °C and used for DNA manipulations as well as for the expression of toxin variants.

### Construction of plasmids for production of CyaA and HlyA variants

Site-directed PCR mutagenesis was employed to generate single or multiple substitutions within the *cyaA* and *hlyA* genes. The mutagenized *cyaA* PCR fragments were inserted into the parental *cyaA* sequence in the pT7CACT1 construct (31), while the mutagenized *hlyA* fragments were inserted into the parental *hlyA* sequence in pT7*hlyC*-*hlyA* (56). Oligonucleotide-based PCR mutagenesis was applied to generate constructs derived from pT7CT7ACT1-sHis-TEV to produce unacylated AS-RTX variants (WT, N936L, G934L and D880L), in which the C-terminal folding scaffold of RTX block V of CyaA (residues 1562–1681) was fused to CyaA segments comprising residues 734 to 1038. All final plasmid constructs were confirmed by DNA sequencing performed by Eurofins Genomics.

### Production and purification of CyaA and HlyA variants

CyaA and HlyA variants were produced in *E. coli* XL1-Blue cells transformed with the appropriate plasmids and purified from urea extracts as previously described (56, 57). Urea extracts containing AS-RTX proteins were applied to Ni-NTA agarose columns equilibrated with TUN buffer (50 mM Tris-HCl (pH 8.0), 8 M urea, and 300 mM NaCl). After washing with the same buffer, the AS-RTX variants were eluted using TUN buffer supplemented with 250 mM imidazole. Protein purity was assessed by SDS-PAGE, protein concentrations were quantified by the Bradford assay (Bio-Rad), and all proteins were stored at −20 °C. Purification and storage were performed in buffers containing 8 M urea to maintain the proteins in a stable, non-aggregated state.

Prior to functional assays on eukaryotic cells, the purified denatured CyaA and HlyA variants were refolded from buffers containing 8 M urea by rapid 100-fold dilution into appropriate Ca^2+^-containing buffers and used immediately. This procedure is important for achieving maximal biological activity, as it mimics the physiological mechanism during type I secretion, where RTX toxins emerge from the secretion apparatus in a Ca^2+^-free, unfolded state and fold rapidly upon exposure to millimolar extracellular Ca^2+^ (32).

For hydrogen/deuterium exchange mass spectrometry (HDX-MS) analysis, CyaA was first concentrated to a final stock concentration of 20 mg/ml (∼113 μM) using Amicon ultrafiltration units with a 10 kDa cutoff membrane (MilliporeSigma) and then diluted to various final urea concentrations down to 0.4 M, at which the toxin was found to be folded (Fig. S8*A*). Dilutions to lower urea concentrations were constrained by the maximum attainable starting CyaA concentration (∼113 μM) and the minimum toxin concentration (5 μM) required for robust HDX-MS analysis. Consequently, this 20-fold dilution (final 0.4 M urea) was also consistently used in CD and thermal stability assays to maintain the toxin variants in a stable, folded state throughout the measurements.

### Determination of adenylate cyclase activity

AC activity was measured using a previously reported procedure (58). One unit of AC activity was defined as the amount of protein that generates 1 μmol of cAMP per minute at 30 °C and pH 8.0.

### Cell binding and cell invasive activities of CyaA on erythrocytes

Sheep erythrocytes (LabMediaServis, Jaroměř, Czech Republic) were washed with TCN buffer (50 mM Tris-HCl (pH 7.4), 2 mM CaCl_2_, and 150 mM NaCl), adjusted with TCN buffer to 5 × 10^8^ cells/ml, and incubated with CyaA or its variants for 30 min at 37 °C (51). One aliquot of cells was extensively washed in cold TEN buffer (50 mM Tris-HCl (pH 7.4), 5 mM EDTA, and 150 mM NaCl) and used to assess cell-bound AC activity (binding). Another aliquot was treated with trypsin (20 μg/ml, 15 min, 37 °C) to digest the extracellular AC domain. Trypsin inhibitor (40 μg/ml) was then added, and the erythrocytes were washed twice with cold TEN buffer before being used to determine intracellular AC activity (invasive intracellular AC).

### Hemolytic activity

Hemolytic activity was determined at 37 °C in TCN buffer (pH 7.4) by measuring absorbance at 541 nm (A_541_), which reflects hemoglobin release from erythrocytes (5 × 10^8^ cells/ml) (51).

### Cell line

Human monocytic/macrophage THP-1 cells (ATTC number TIB-202) were cultured in RPMI medium supplemented with penicillin (100 I.U./ml), streptomycin (100 μg/ml), amphotericin B (250 ng/ml), and 10% heat-inactivated fetal bovine serum. Prior to assays, RPMI was substituted with serum-free Dulbecco's Modified Eagle Medium (DMEM) containing 1.9 mM Ca^2+^.

### Binding of CyaA to THP-1 cells

THP-1 cells (1 × 10^6^/ml) were incubated with CyaA or its variants (1 μg/ml) in DMEM for 30 min at 4 °C (51). Unbound toxin was removed by washing the cells three times with DMEM. The cells were then lysed using 0.1% Triton X-100, and AC activity associated with cell membranes was quantified as described above.

### cAMP determination

THP-1 cells (1.5 × 10^5^) in DMEM were incubated with CyaA or its variants for 30 min at 37 °C. To terminate the reaction, 0.2% Tween-20 in 100 mM HCl was added, and the samples were boiled at 100 °C for 15 min. After neutralization with 150 mM unbuffered imidazole, intracellular cAMP levels were quantified by a competitive immunoassay as previously described (59).

### Cell viability assay

Viability of THP-1 cells in the presence of HlyA or its variants was assessed in DMEM using the WST-1 assay kit (Roche) as previously described (51).

### Mass spectrometric quantification of toxin acylation

The acylation status of proteins was analyzed by high-resolution liquid chromatography-mass spectrometry (LC-MS) as described earlier (51). Briefly, purified CyaA and HlyA toxins and their mutant variants in 8 M urea were normalized to the final concentration of 0.5 mg/ml, and the buffer conditions of 4 M urea, 10% acetonitrile (v/v), 50 mM N-ethylmorpholine/acetate, pH 8.3, and subsequently digested for 2 h at 30 °C with LysC (1/40 enzyme:substrate ratio; Lysyl Endopeptidase, FUJIFILM Wako) and an additional 4 h at 30 °C with trypsin (1/40 enzyme:substrate ratio; Sequencing Grade Modified Trypsin, Promega). Digestion was quenched with trifluoroacetic acid (Merck) to reach a 0.1% final concentration. Resulting peptides were diluted 1:1 in 0.1% FA (v/v), and 1.25 μg equivalents were loaded on the C18 trap column (20 × 0.30 mm, Luna Omega 5 μm Polar C18 100 Å, Phenomenex) and subsequently separated on an analytical column (150 × 0.3 mm, Luna Omega 3 μm Polar C18 100 Å, Phenomenex). Chromatographic separation was carried out on Agilent 1290 binary pump and 1260 linear pump (Agilent Technologies), using a linear gradient of 5 to 65% solution B (70% acetonitrile, 25% isopropanol, 0.16% formic acid) over solution A (2.5% acetonitrile, 2.5% isopropanol, 0.2% formic acid) at a flow rate of 10 μl/min over 34 min and a linear gradient of 65 to 95% for an additional 1 min. LC-MS/MS data were acquired on a maXis I Q-ToF instrument (Bruker Daltonics) using a data-dependent auto-MS/MS method in positive mode, selecting the five most abundant precursor ions with a cycle time of 3.0 s, mass range from m/z 200 to 1700, and exclusion release of 19 s. All runs were converted from Bruker.d to mzML format using Proteo Wizard MSConvert (v3.0.25178–521213f (automated build)) [https://doi.org/10.1038/nbt.2377], and processed with custom mass offsets with MSFragger (v4.3), and IonQuant (v1.11.11) in FragPipe platform (v23.0) [https://doi.org/10.1016/j.mcpro.2021.100077] against a custom database containing CyaA and HlyA WT and mutant variants, and standard contaminant sequences (cRAP.fasta) with 1% FDR value. The FragPipe settings were as follows: K/R protease specificity, two missed cleavages, no fixed modification and stearoylation, oleoylation, linoleoylation, linolenoylation, palmitoleoylation, juniperoylation, palmitoylation, hydroxymyristoylation, myristoylation, myristoleoylation variable modifications. Quantitation was done based on the intensity of the first isotope at the maximum of the EIC in Bruker Compass DataAnalysis software v6.1 (Bruker Daltonics, https://www.bruker.com/en/products-and-solutions/mass-spectrometry/ms-software.html). All files are available on PRoteomics IDentifications Database.

### CD spectroscopy

Far-UV CD spectra were recorded at 25 °C using a Chirascan-plus spectrometer (Applied Photophysics) in rectangular quartz Suprasil cuvettes with a 1 mm path length (110-QS, Hellma). Urea-unfolded protein samples (2 mg/ml) were refolded by rapid 20-fold dilution to a final concentration of 0.1 mg/ml in 5 mM Tris-HCl (pH 8.0) and 20 mM NaCl, either in the absence or presence of 2 mM CaCl_2_. Immediately after dilution, the spectra were collected over a wavelength range of 200 to 260 nm at a scan speed of 1 nm/s. Buffer spectra were subtracted from the corresponding protein spectra, and molar ellipticity was expressed in degrees square centimeter per decimole [deg.cm^2^.dmol^-1^] as previously described (19). For each protein variant, two independently prepared samples were analyzed. For each sample, CD spectra were recorded in three independent measurements, and each spectrum represented the average of three consecutive scans.

### Thermal stability

Thermal stability assays were performed using nanoDSF on a Prometheus NT.48 instrument (NanoTemper Technologies). Urea-unfolded proteins (2 mg/ml) were refolded by rapid 20-fold dilution to a final concentration of 0.1 mg/ml in TNC buffer (pH 8.0) and immediately loaded into nanoDSF grade standard capillaries (NanoTemper Technologies). Measurements were carried out from 25 to 95 °C with a temperature ramp of 1.5 °C/min, while continuously monitoring tryptophan fluorescence at 350 and 330 nm. The melting temperature, corresponding to the inflection point of the unfolding curve, was determined by using a PR.ThermControl (NanoTemper Technologies), as previously described (19). For each CyaA variant, two independent protein preparations were analyzed across a total of three experiments.

### Planar lipid bilayers

Planar lipid bilayer measurements were conducted as previously described (51). Briefly, L-α-phosphatidylcholine (type II-S, Sigma-Aldrich) was dissolved in n-decane–butanol (9:1, vol/vol) and applied to a circular aperture in a diaphragm separating two Teflon chambers, each filled with 10 mM Tris-HCl (pH 7.4), 2 mM CaCl_2_, and 150 mM KCl. Membrane currents were recorded at 25 °C using Ag/AgCl electrodes under an applied voltage of -50 mV, amplified by LCA-200 to 10G and LCA-200 to 100G amplifiers (Femto), and digitized using a LabQuest Mini A/D converter (Vernier).

### Hydrogen/deuterium exchange mass spectrometry (HDX-MS)

To study the effect of different urea concentrations on the folding of CyaA, HDX-MS was performed. Ca^2+^-mediated folding was initiated by diluting the toxin stock into a folding buffer consisting of 50 mM Hepes (pH 7.4), 150 mM NaCl, and 4 mM CaCl_2_, adjusting the urea concentration to 8 M, 4 M, 2.4 M, 1.6 M, 0.8 M, and 0.4 M. A subsequent dilution step was performed to reach a final protein concentration of 5 μM without altering the buffer composition. The folding was allowed to proceed for 1 and 16 h prior to initiating deuterium exchange. Samples were then centrifuged to remove any potential aggregates; no aggregation was observed, and no decrease in protein concentration was detected. Next, HDX-MS was used to assess the structural impact of the D880L mutation in CyaA. Protein solution was prepared following the same procedure as described above, with adjusting the urea concentration to either 2.4 M or 1.6 M. The folding was allowed to proceed for 1, 8, or 16 h prior to initiating deuterium exchange. Deuterium exchange was carried out by diluting the folded protein solution five-fold into a D_2_O-based buffer that matched the urea concentration used. Labeling was conducted at 21 °C across five-time intervals: 20 s, 1 min, 5 min, 20 min, and 2 h. Each condition was analyzed in triplicate, and an additional 20 s labeling time was included at the end of each series to monitor CyaA stability. Exchange reactions were stopped by mixing the sample with an equal volume of ice-cold quench solution (1 M glycine-HCl, pH 2.3) containing 0 M, 4 M, 5.6 M, 6.4 M, 7.2 M, or 7.6 M urea, depending on the exchange condition.

All sample handling steps, including folding, labeling, and quenching, were automated using a PAL DHR system (CTC Analytics) operated *via* Chronos software (AxelSemrau, Sprockhövel, https://www.axelsemrau.de/en/productdetails/chronos). Immediately after quenching, samples were introduced into a UPLC system maintained at 0 °C to suppress back-exchange. Proteins were digested online using an immobilized pepsin column (69 μl bed volume), followed by peptide trapping on a SecurityGuard ULTRA Polar C18 cartridge (2.1 mm ID, Phenomenex). Peptide separation was achieved on a Luna Omega Polar C18 analytical column (1.6 μm, 100 Å, 1.0 × 100 mm, Phenomenex). Desalting was performed using 0.4% formic acid in water at a flow rate of 100 μl/min, delivered by an Agilent 1260 Infinity II quaternary pump (Agilent Technologies). Peptides were eluted *via* a linear acetonitrile gradient (10% to 45%) followed by a final step to 99% solvent B (0.1% formic acid in 98:2 acetonitrile:water), using a 1290 Infinity II binary pump (Agilent Technologies) at 40 μl/min.

Mass spectrometric analysis was performed on a timsTOF Pro (Bruker Daltonics) operated in the MS1 mode at an acquisition rate of 1 Hz, with ion mobility separation disabled. Raw data were processed using DataAnalysis (Bruker Daltonics) and DeutEx. Data visualization was carried out with MSTools and PyMOL (https://www.pymol.org/).

To identify peptides arising from online pepsinolysis, data-dependent MS/MS acquisition with PASEF was performed using the same LC-MS configuration as described above. Spectra were analyzed with MASCOT (v2.7, Matrix Science, https://www.matrixscience.com/) against a custom database containing CyaA WT and D880L variant, pepsin, and standard contaminant sequences (cRAP.fasta). Search parameters included a precursor mass tolerance of 10 ppm, fragment ion tolerance of 0.05 Da, a minimum peptide length of six amino acids, and an IonScore threshold above 20. A false discovery rate of less than 1% was enforced. Variable modifications on lysine residues included a panel of acyl groups: stearoyl, palmitoyl, myristoyl, myristoyl-OH, myristoleyl, stearoyl 18:1, and palmitoleyl. All experiments were performed as two independent biological replicates, each including technical replication of folding (HDX started after 1, 8 and 16 h post-folding) and HDX-MS analysis.

### Structure prediction

Structural models of HlyA, RtxA, and ApxIA were predicted using AlphaFold2 (v. 2.2.0) with the standard AlphaFold pipeline (60). Multiple sequence alignments were generated using the reduced database preset (--db_preset = reduced_dbs), including UniRef90, MGnify, and small BFD databases. Template use was enabled with a template date restriction (--max_template_date = 2020–05–14) to exclude the cryoEM structure of the CyaA RTX751 fragment (33). Amber relaxation was enabled for structural refinement. Predictions were performed using three recycles, and five models were generated for each protein.

### Statistical analysis

Results are presented as the arithmetic mean ± SD. Statistical significance was determined by one-way ANOVA followed by Dunnett's test for *post hoc* analysis, and Fig. S6 was tested by an unpaired, 2-tailed Student's *t* test (GraphPad Prism 10.2.2; GraphPad Software).

## Data availability

Proteomics data from mass spectrometry analyses were deposited to the ProteomeXchange Consortium (61) through the PRIDE repository (http://www.ebi.ac.uk/pride) (62) under the identifiers PXD058094 and PXD065966. Additional datasets generated in this study can be obtained from the corresponding authors upon reasonable request.

## Supporting information

This article contains supporting information.

## Conflict of interest

The authors declare that they have no conflicts of interest with the contents of this article.
