# Supplementary material for: Calcium-loaded acylated segment controls membrane penetration capacity of repeats-in-toxin cytolysins
Source: J Biol Chem. 2026 Apr 27;302(6):113079. doi: 10.1016/j.jbc.2026.113079 (PMC13224072; doi:10.1016/j.jbc.2026.113079)
Supplement: Supplementary material [file mmc1.pdf]

Supporting Information

Calcium-loaded acylated segment controls membrane penetration capacity of repeats-in-toxin cytolysins

Jiri Masin[1,*], Adriana Osickova[1], Zuzana Kalaninova[1,2], Petr Man[1], Ladislav Bumba[1], Sascha Vatic[1], Petr Novak[1], Michaela Buresova[1,2], Anna Lesniak[1,2], Joana Filipa Tinoco Marçal[1], David Jurnecka[1], Humaira Khaliq[1], Peter Sebo[1], and Radim Osicka[1,*]

[1]Institute of Microbiology of the Czech Academy of Sciences, Prague, Czech Republic

[2]Faculty of Sciences, Charles University, Prague, Czech Republic

[*] Corresponding author

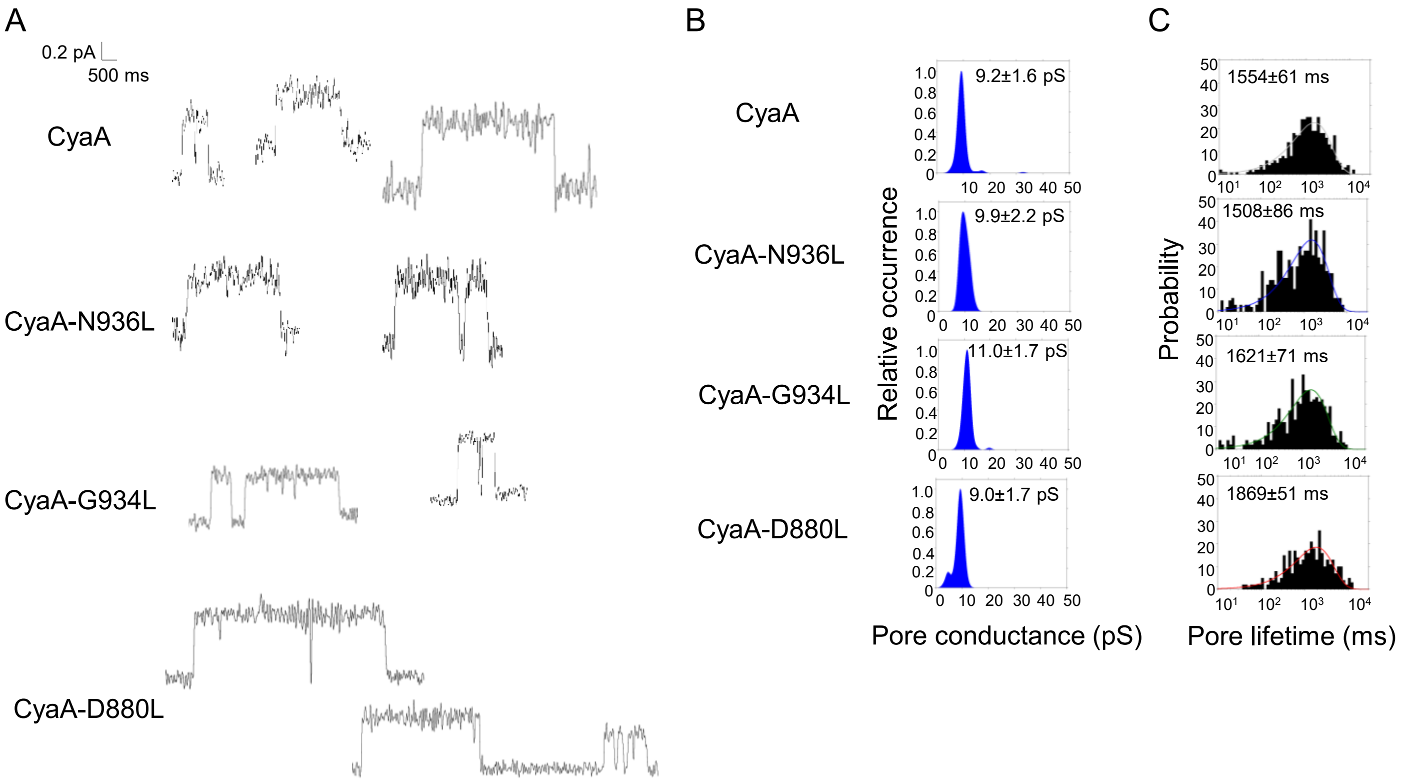

Figure S1: **Pore characteristics of CyaA variants on black lipid membranes are similar. (A)** Recording of single pore conductance occurring in asolectin membranes exposed to 10 pM CyaA variants and bathing in 50 mM KCl, 10 mM Tris–HCl (pH 7.4), 2 mM $CaCl_2$, with -50 mV applied voltage at 25 °C. The recordings were filtered at 10 Hz. **(B)** Kernel density estimation of single-pore conductances was calculated from over 300 events acquired on several asolectin membranes with 5-10 pM CyaA under the same conditions as in Fig. 2C. The numbers represent the most frequent conductances ± standard deviations of pores formed by the CyaA variants. **(C)** Lifetime determination of ~300 individual pore openings recorded on several different asolectin membranes with 5-10 pM CyaA or its variants under the same conditions as in Fig. 2C. Error estimates of lifetimes were obtained by bootstrap analysis, with the most frequent values ± standard deviations shown.

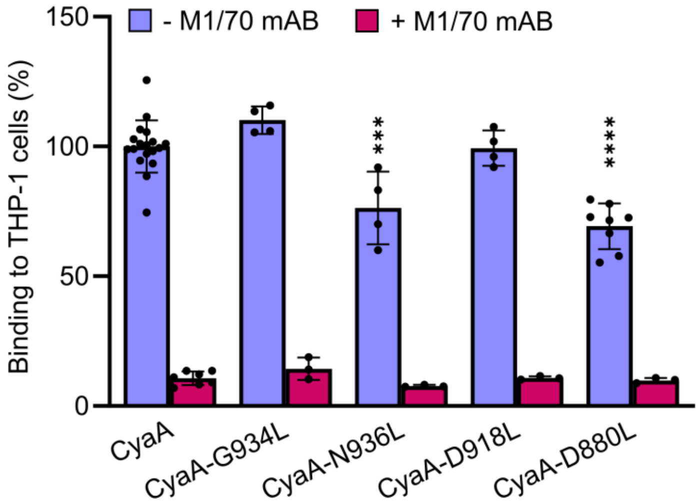

Figure S2. Binding of CyaA variants to THP-1 cells depends on CR3. The binding of intact CyaA or its mutant variants to THP-1 cells (1×10^6) was determined in D-MEM medium as the amount of total cell-associated AC enzyme activity after incubation of cells with 1 μg/ml of the protein for 30 min at 4°C. To block the CR3, THP-1 cells were incubated on ice with 5 μg/ml of the CD11b-specific monoclonal antibody M1/70 (BD Pharmingen, Franklin Lakes, USA) for 15 min prior to addition of the CyaA variants. Activities are expressed as percentages of the intact CyaA activity. Data represent mean values from at least three independent experiments. Statistical significance was determined by the one-way ANOVA. ****, $p<0.0001$, ***, $p<0.001$.

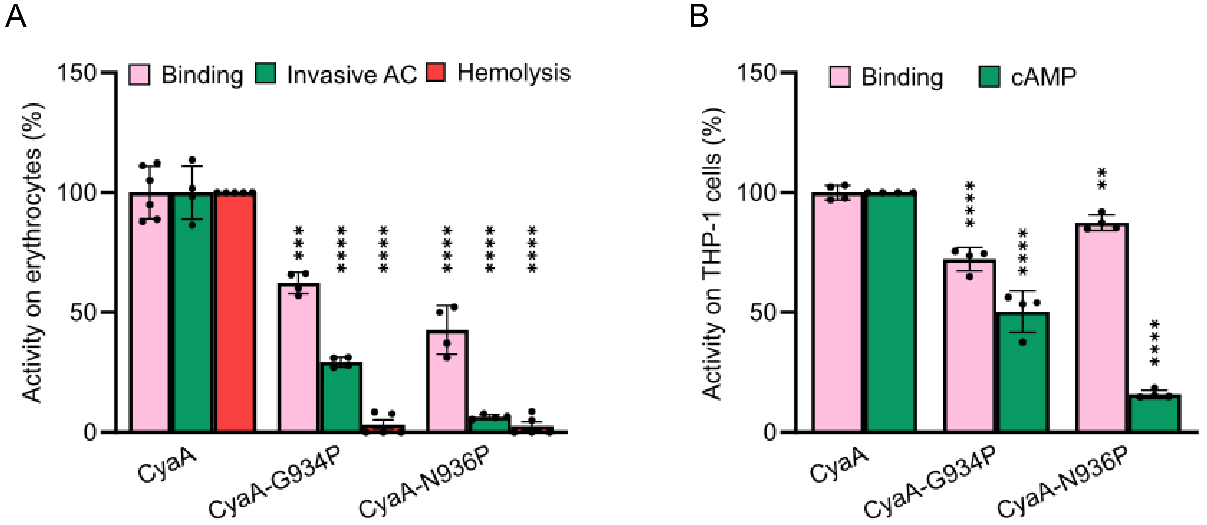

Figure S3. Activities of CyaA variants on erythrocytes and THP-1 cells. (A) Cell binding and cell invasive AC activities of CyaA variants were assessed on sheep erythrocytes (5 × 10^8/ml) exposed to 1 μg/ml of the protein in 50 mM Tris-HCl (pH 7.4), 150 mM NaCl, 2 mM $CaCl_2$ (TCN buffer) for 30 min at 37 °C. Hemolytic activity was assessed on sheep erythrocytes (5 × 10^8/ml in TCN buffer) exposed for 4 h to 5 μg/ml of the protein at 37 °C as A_{541nm}. Activity of intact CyaA was taken as 100%. Data represent mean values from al least three independent experiments performed in duplicate, using two independent toxin preparations. **(B)** Binding of CyaA variants to THP-1 cells (1 × 10^6) was determined as the amount of total cell-associated AC enzyme activity upon incubation of cells in D-MEM (1.9 mM Ca^{2+}) with 1 μg/ml of the protein for 30 min at 4 °C. cAMP intoxication was assessed by determining the intracellular concentration of cAMP generated in cells at 37 °C after 30 min of incubation of THP-1 cells (1.5 × 10^5 in D-MEM medium) with different CyaA concentrations from within the linear range of the dose-response curve (250 to 15.5 ng/ml). Activities are expressed as percentages of intact CyaA activity. Data represent mean values from at least three independent determinations performed in duplicate, using two different toxin preparations. Statistical significance was determined by the one-way ANOVA. ****, p<0.0001, ***, p<0.001, **, p<0.01.

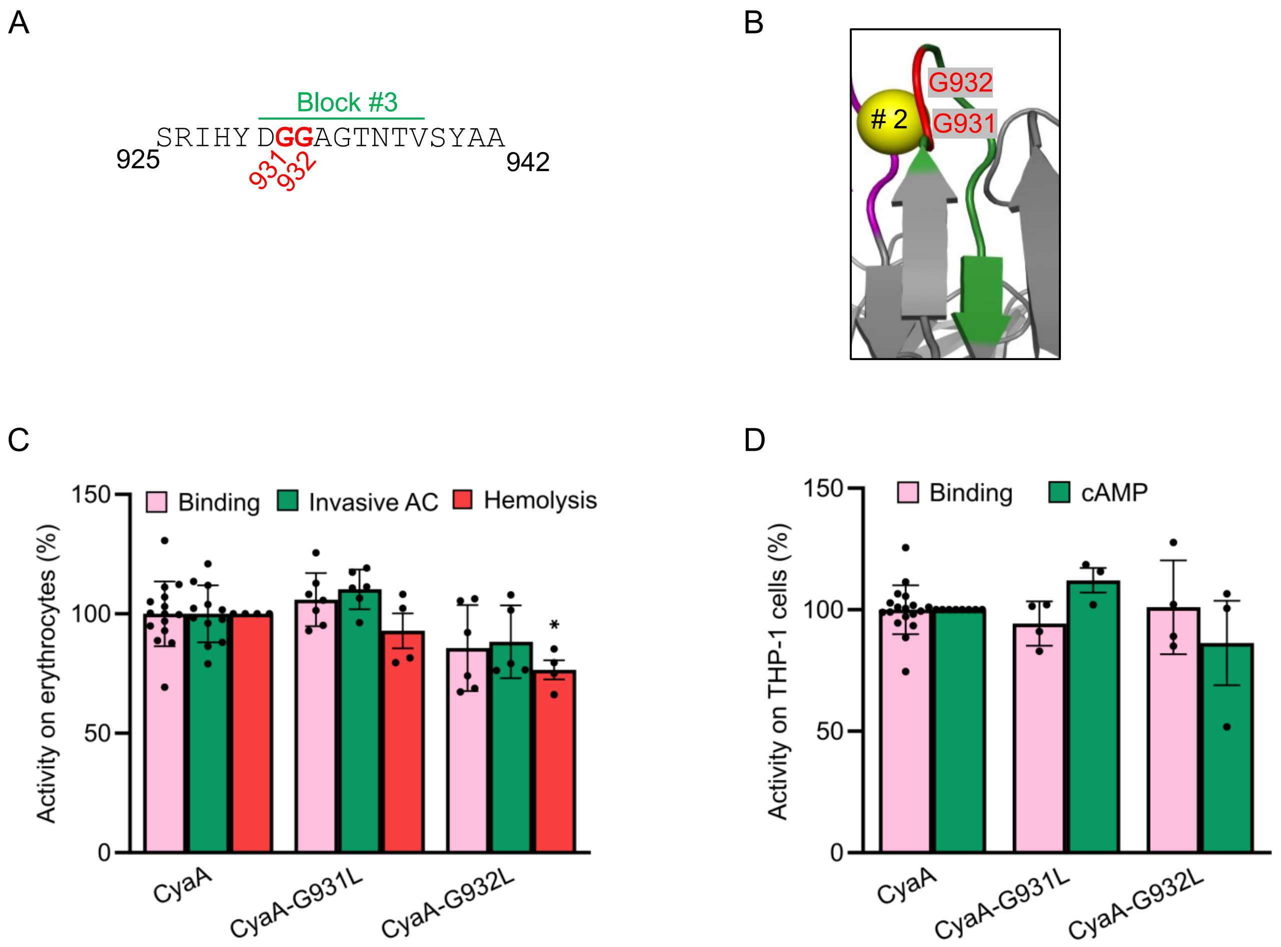

Figure S4. Activities of CyaA variants on erythrocytes and THP-1 cells. (A) The G931 and G932 residues from block #3 are shown in red. **(B)** PyMOL view of RTX751 fragment with block #3 of Gly- and Asp/Asn-rich nonapeptides (in green) involved in Ca^{2+} binding. Ca^{2+} ion #2 is depicted by yellow sphere. **(C)** Cell binding, cell invasive AC, and hemolytic activity of CyaA variants were assessed on sheep erythrocytes as described in detail in Fig. S3. **(D)** Activities on THP-1 cells were determined as described in Fig. S3. Activities are expressed as percentages of intact CyaA activity. Data represent mean values from at least three independent determinations performed in duplicate, using two different toxin preparations. Significance of differences was determined using the one-way ANOVA. *, $p<0.05$.

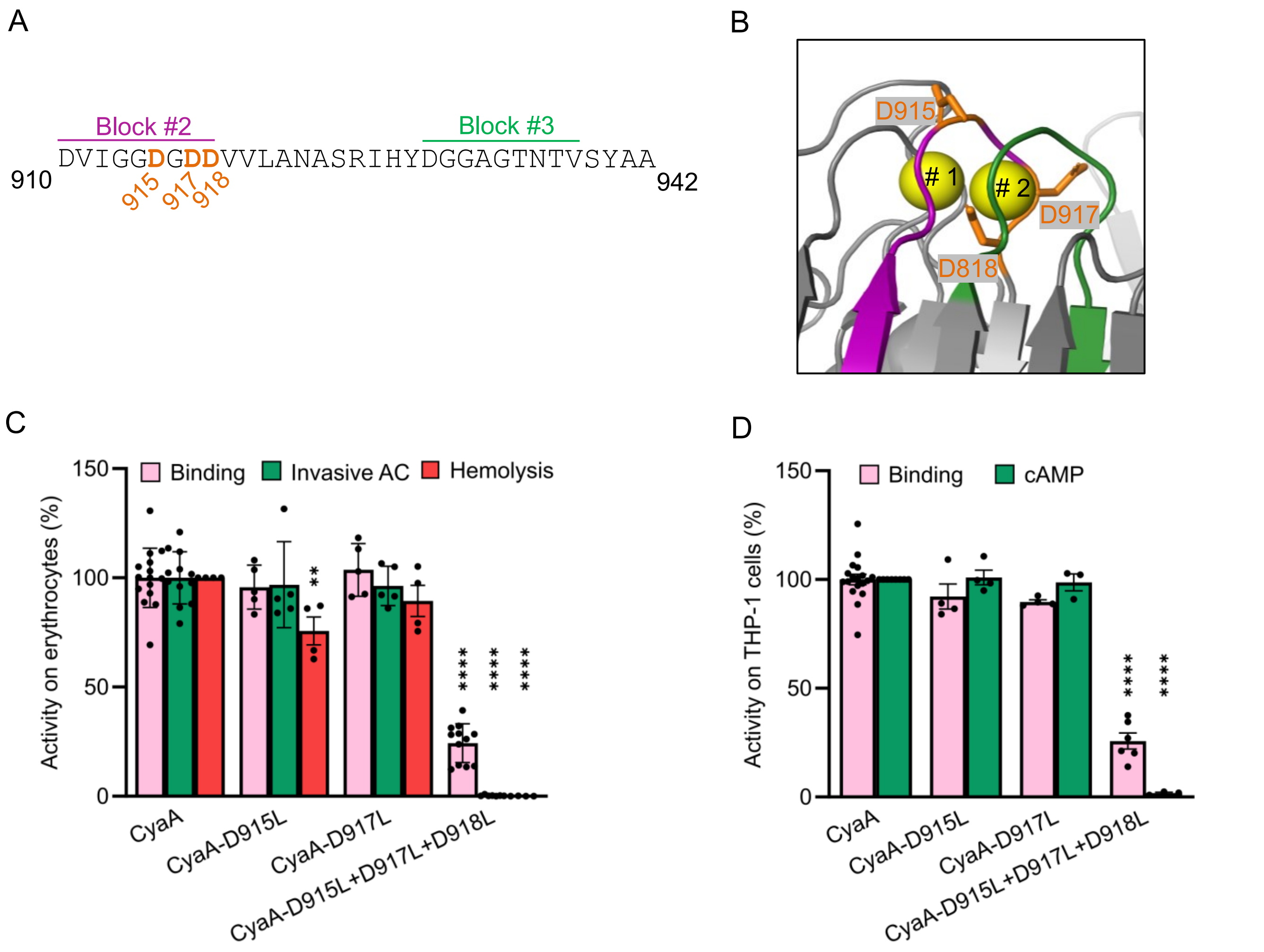

Figure S5. Activities of CyaA variants on erythrocytes and THP-1 cells. (A) The Gly and Asp/Asn-rich sequences within the acylated domain of CyaA are highlighted in green (block #3) and violet (block #2), residues D915, D917, and D918 are in orange. **(B)** PyMOL view of RTX751 fragment with two blocks of Gly and Asp/Asn-rich nonapeptides (labelled in violet and green) involved in Ca^{2+} binding. Ca^{2+} ions #1 and #2 are depicted by yellow spheres. **(C)** Cell binding, cell invasive AC, and hemolytic activity of CyaA variants were assessed on sheep erythrocytes as described in detail in Fig. S3. Activity of intact CyaA was taken as 100%. **(D)** Activities on THP-1 cells were determined as described in Fig. S3. Activities are expressed as percentages of intact CyaA activity. Data represent mean values from at least three independent determinations performed in duplicate, using two different toxin preparations. Significance of differences was determined using the one-way ANOVA. ****, p<0.0001, **, p<0.01.

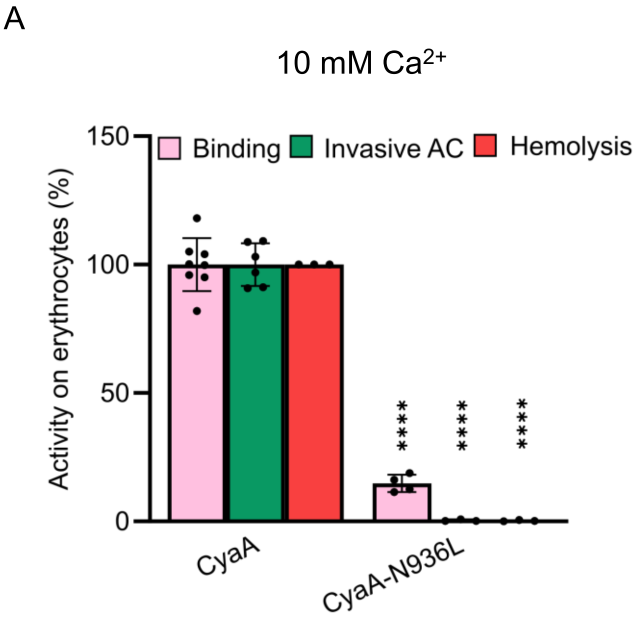

Figure S6. Activities of CyaA variants on erythrocytes and THP-1 cells in 10 mM Ca^{2+}. Toxin activities on **(A)** sheep erythrocytes in TN buffer (50 mM Tris, 150 mM NaCl, pH 7.4) supplemented with 10 mM $CaCl_2$, or on **(B)** THP-1 monocytes in modified HBSS buffer (140 mM NaCl, 5 mM KCl, 3 mM $MgCl_2$, 10 mM HEPES-Na pH 7.4, 50 mM glucose, 10 mM $CaCl_2$) were analyzed as described in detail in Fig. S3. Activities are expressed as percentages of intact CyaA activity. Data represent mean values from at least three independent determinations performed in duplicate, using two different toxin preparations. Statistical significance was determined using the Student´s t-test. ****, $p<0.0001$.

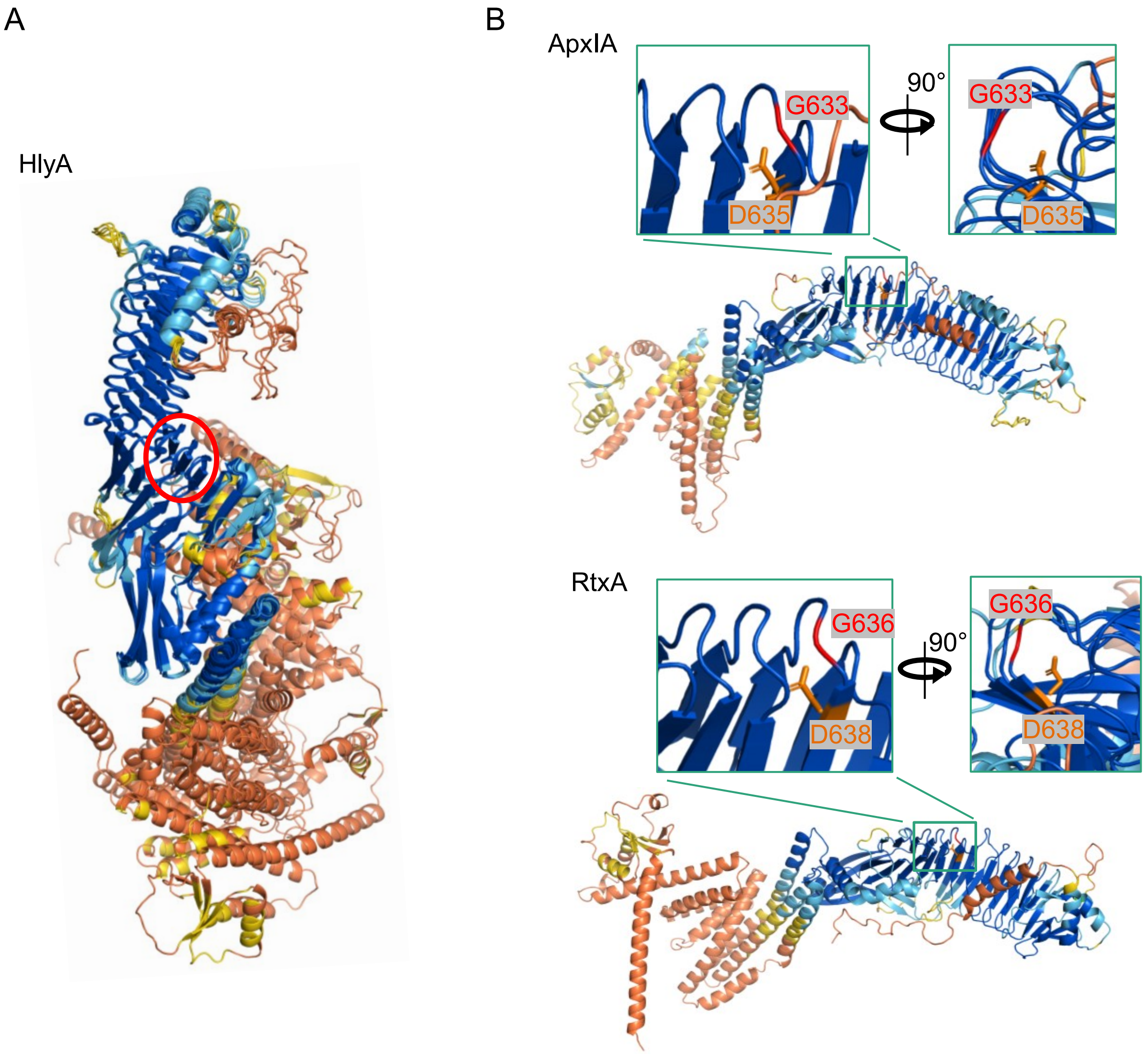

Figure S7. (A) An overlay of the three AlphaFold2 models of *E. coli* HlyA shown in colors representing a per-residue confidence score (pLDDT). Blue: very high (pLDDT > 90), light blue: confident (90 > pLDDT > 70), yellow: low (70 > pLDDT > 50), orange: very low (pLDDT < 50). The possible binding site for Ca^{2+} ions in the acylated segment is marked with a red circle. **(B)** AlphaFold2 models of ApxIA from *Actinobacillus pleuropneumoniae* (UniProtKB P55128) and RtxA from *Kingella kingae* (UniProtKB A0A1X7QMH9). Aspartate residues hypothetically involved in Ca^{2+} ion coordination are labeled in orange, and the position of the glycine residue is shown in red. Models are colored by pLDDT, as described in panel A.

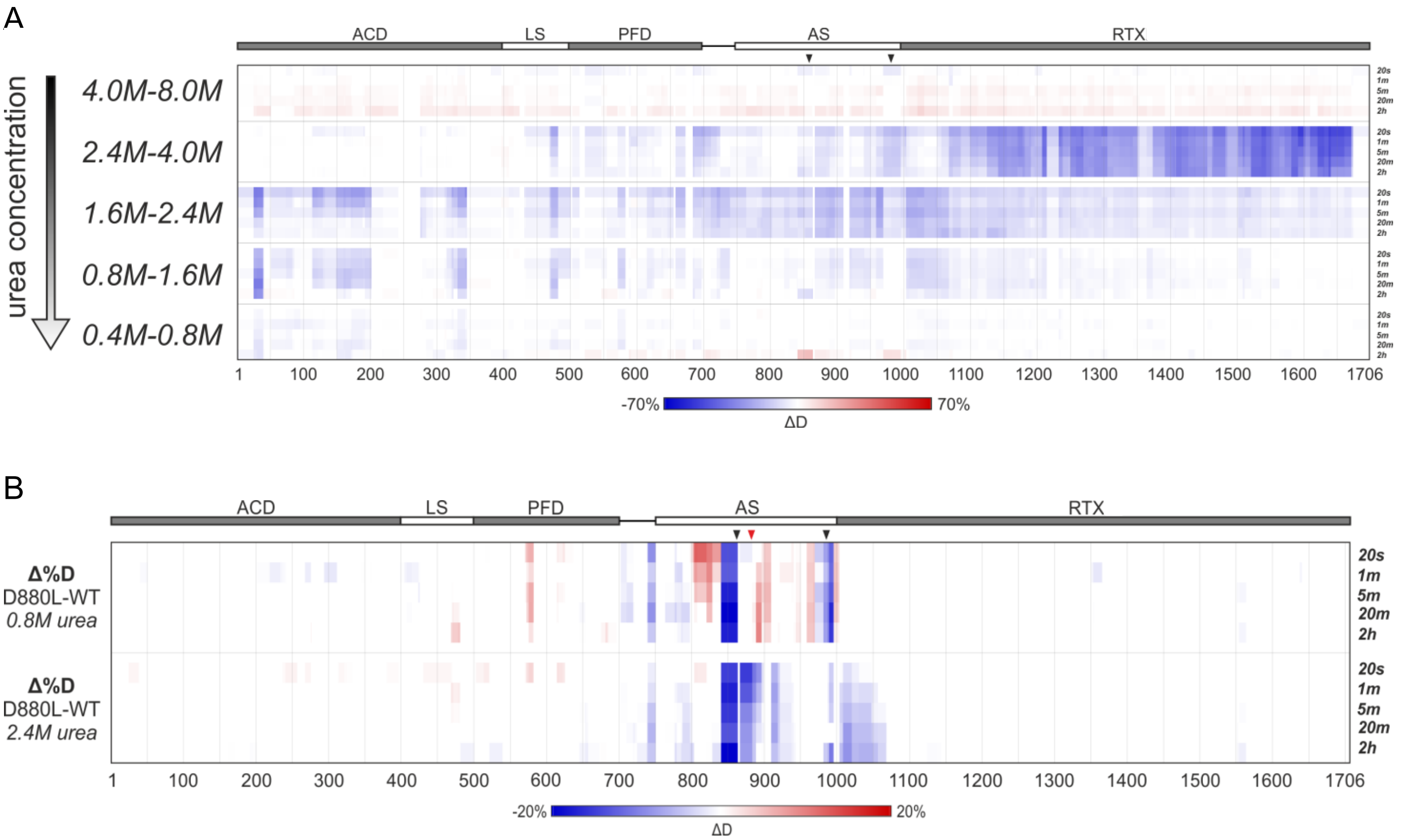

Figure S8. (A) Individual folding steps of CyaA visualized *via* differential heat maps. Deuteration differences between two consecutive urea concentration steps are presented as a blue-white-red gradient, where blue indicates lower deuteration and red indicates higher deuteration. This image captures the main folding transition of the C-terminal half between 4 and 2.4 M urea, followed by the compaction of the middle and N-terminal parts between 2.4 and 1.6 M urea, and culminating in the folding of the N-terminus between 1.6 and 0.8 M urea. At this concentration, the protein is stable, as a further decrease in urea concentration is not associated with structural compaction of any part of CyaA. Toxin domains and positions of acylation sites (black arrowheads) are annotated above the heat maps. Regions not covered by HDX-MS data are represented in white. **(B) Heat maps for the entire CyaA sequence, showing the differences in deuteration levels between wild-type CyaA (WT) and the D880L mutant at 0.8 M and 2.4 M urea concentration.** A blue (protection/lower deuteration)-white (no change)-red (deprotection/higher deuteration) color gradient was used to represent the changes. Domains, acylation sites (black arrowheads) and D880 position (red arrowhead), are shown above the heat map. ACD, AC domain; LS, linker segment; PFD, pore-forming domain, AS, acylated segment; RTX, RTX domain.

Table S1. Melting temperature values of CyaA variants

Protein[a]	T_m (°C)[b]
CyaA	63.6 ± 0.80
CyaA-D880L	63.4 ± 0.84
CyaA-N936L	63.6 ± 0.45
CyaA-G934L	64.1 ± 0.67

[a] Proteins were produced in the *E. coli* strain XL-1 Blue and purified as described in Experimental Procedures.

[b] Melting temperature (T_m) values, corresponding to the inflection points of the unfolding curves, were performed by nanoDSF. Average T_m values ± standard deviations from analysis of two independent toxin preparations are shown (N=4).
